# Potential of therapeutic bile acids in the treatment of neonatal Hyperbilirubinemia

**DOI:** 10.1038/s41598-021-90687-5

**Published:** 2021-05-27

**Authors:** Lori W. E. van der Schoor, Henkjan J. Verkade, Anna Bertolini, Sanne de Wit, Elvira Mennillo, Eva Rettenmeier, André A. Weber, Rick Havinga, Petra Valášková, Jana Jašprová, Dicky Struik, Vincent W. Bloks, Shujuan Chen, Andrea B. Schreuder, Libor Vítek, Robert H. Tukey, Johan W. Jonker

**Affiliations:** 1 Section of Molecular Metabolism and Nutrition, Laboratory of Pediatrics, University of Groningen, University Medical Center Groningen, Hanzeplein 1, 9713 GZ Groningen, The Netherlands; 2 Pediatric Gastroenterology and Hepatology, University of Groningen, University Medical Center, Hanzeplein 1, 9713 GZ Groningen, The Netherlands; 3 Laboratory of Environmental Toxicology, Department of Pharmacology, University of California, San Diego, La Jolla, CA 92093 USA; 4 Fourth Department of Internal Medicine and Institute of Medical Biochemistry and Laboratory Diagnostics, First Faculty of Medicine, Charles University, Prague, Czech Republic

**Keywords:** Gastrointestinal models, Paediatric research

## Abstract

Neonatal hyperbilirubinemia or jaundice is associated with kernicterus, resulting in permanent neurological damage or even death. Conventional phototherapy does not prevent hyperbilirubinemia or eliminate the need for exchange transfusion. Here we investigated the potential of therapeutic bile acids ursodeoxycholic acid (UDCA) and obeticholic acid (OCA, 6-α-ethyl-CDCA), a farnesoid-X-receptor (FXR) agonist, as preventive treatment options for neonatal hyperbilirubinemia using the hUGT1*1 humanized mice and *Ugt1a*-deficient Gunn rats. Treatment of hUGT1*1 mice with UDCA or OCA at postnatal days 10–14 effectively decreased bilirubin in plasma (by 82% and 62%) and brain (by 72% and 69%), respectively. Mechanistically, our findings indicate that these effects are mediated through induction of protein levels of hUGT1A1 in the intestine, but not in liver. We further demonstrate that in *Ugt1a*-deficient Gunn rats, UDCA but not OCA significantly decreases plasma bilirubin, indicating that at least some of the hypobilirubinemic effects of UDCA are independent of UGT1A1. Finally, using the synthetic, non-bile acid, FXR-agonist GW4064, we show that some of these effects are mediated through direct or indirect activation of FXR. Together, our study shows that therapeutic bile acids UDCA and OCA effectively reduce both plasma and brain bilirubin, highlighting their potential in the treatment of neonatal hyperbilirubinemia.

## Introduction

Unconjugated bilirubin (UCB) is a potentially neurotoxic metabolite of heme catabolism. Severe unconjugated hyperbilirubinemia is associated with the development of kernicterus, resulting in permanent neurological damage or even death^1^. Unconjugated hyperbilirubinemia occurs primarily in newborns since they display an increased bilirubin production and a decreased disposal, caused by a combination of increased erythrocyte breakdown and very low or absent expression of the bilirubin conjugation enzyme, UDP-glucuronosyltransferase 1A1 (UGT1A1)^2^. In newborns, bilirubin is also subject to enterohepatic circulation, resulting in substantial intestinal re-absorption and increased systemic exposure^3^. In preterm infants, unconjugated hyperbilirubinemia is especially problematic, since their blood–brain barrier is more permeable and their underdeveloped brain is more susceptible to bilirubin-induced neurotoxicity^4^.

Phototherapy enhances bilirubin disposal from the body and has been the gold standard therapy for unconjugated hyperbilirubinemia for over 60 years^5^. Phototherapy, however, is not always sufficiently effective in infants and does not completely eliminate the need for exchange transfusions^6^. Moreover, phototherapy can only decrease already accumulated UCB but does not prevent its accumulation. In epidemiological studies, phototherapy has also been associated with increased mortality and with the development of infantile cancer^7–11^. Thus, despite the apparent success of phototherapy, there is still a need for alternative or complementary treatments.

The bile acid (BA) ursodeoxycholic acid (UDCA) is commonly used for the treatment of cholestatic liver diseases, and has recently been shown beneficial as an adjuvant treatment for neonatal hyperbilirubinemia^12^. Since we and others have shown that UDCA can successfully decrease plasma bilirubin levels^12,13^, we hypothesized that other anti-cholestatic medications could also be beneficial for unconjugated hyperbilirubinemia. In 2016, obeticholic acid (OCA) was FDA-approved as a treatment for primary biliary cholangitis, for patients in which UDCA does not provide sufficient relief. OCA is a selective agonist of the farnesoid-X-receptor (FXR, NR1H4), a biological sensor for BAs that, among other functions, controls BA homeostasis by modulation of their synthesis and enterohepatic transport^14,15^. In contrast to OCA, UDCA is not a ligand for FXR^16^.

In this study, we used the humanized hUGT1*1 mouse model for unconjugated hyperbilirubinemia. hUGT1*1 mice are transgenic for the entire human *UGT1A* locus, including the *UGT1A1* promoter and the well-described phenobarbital response enhancing module (PBREM)^17^. Because UGT1A1 is expressed at an earlier age in mice as compared to humans, neonatal hyperbilirubinemia normally does not occur in mice^18,19^. Humanized UGT1*1 mice, however, display a similar transcriptional delay in expression of UGT1A1, resulting in severe unconjugated hyperbilirubinemia during the first three weeks of life. After three weeks, the bilirubin levels decrease upon UGT1A1 expression and the mice remain normobilirubinemic during adulthood^17^. Complete knockout of the *Ugt1* locus in mice, when not rescued with expression of the human UGT1 locus, results in death within 4–11 days after birth^17,20^. The expression of the human *UGT1* locus in hUGT1*1 mice rescues the lethality associated with severe neonatal hyperbilirubinemia as a result of the extrahepatic intestinal expression of the human *UGT1A1* gene^17,21^. The physiological inducibility of *hUGT1A1* expression in hUGT1*1 mice in both liver and intestine makes this an attractive model and superior to *Ugt1* deficient models such as the *Ugt1a* knockout mice or Gunn rats, commonly used models for hyperbilirubinemia^22,23^. This inducibility of *hUGT1A1* makes them similar to human neonates, and the only model to study the effects of drugs on human *UGT1A1* expression and bilirubin conjugation. In this study, we assessed the effects of UDCA and OCA on bilirubin levels in plasma and brain in neonatal hUGT1*1 mice. In addition, *Ugt1a* deficient Gunn rats were used as a model to distinguish between UGT1A1-dependent and independent treatment effects.

## Results

### Effect of UDCA and OCA on plasma and brain levels of bilirubin in hUGT1*1 mice

**Figure 1:**
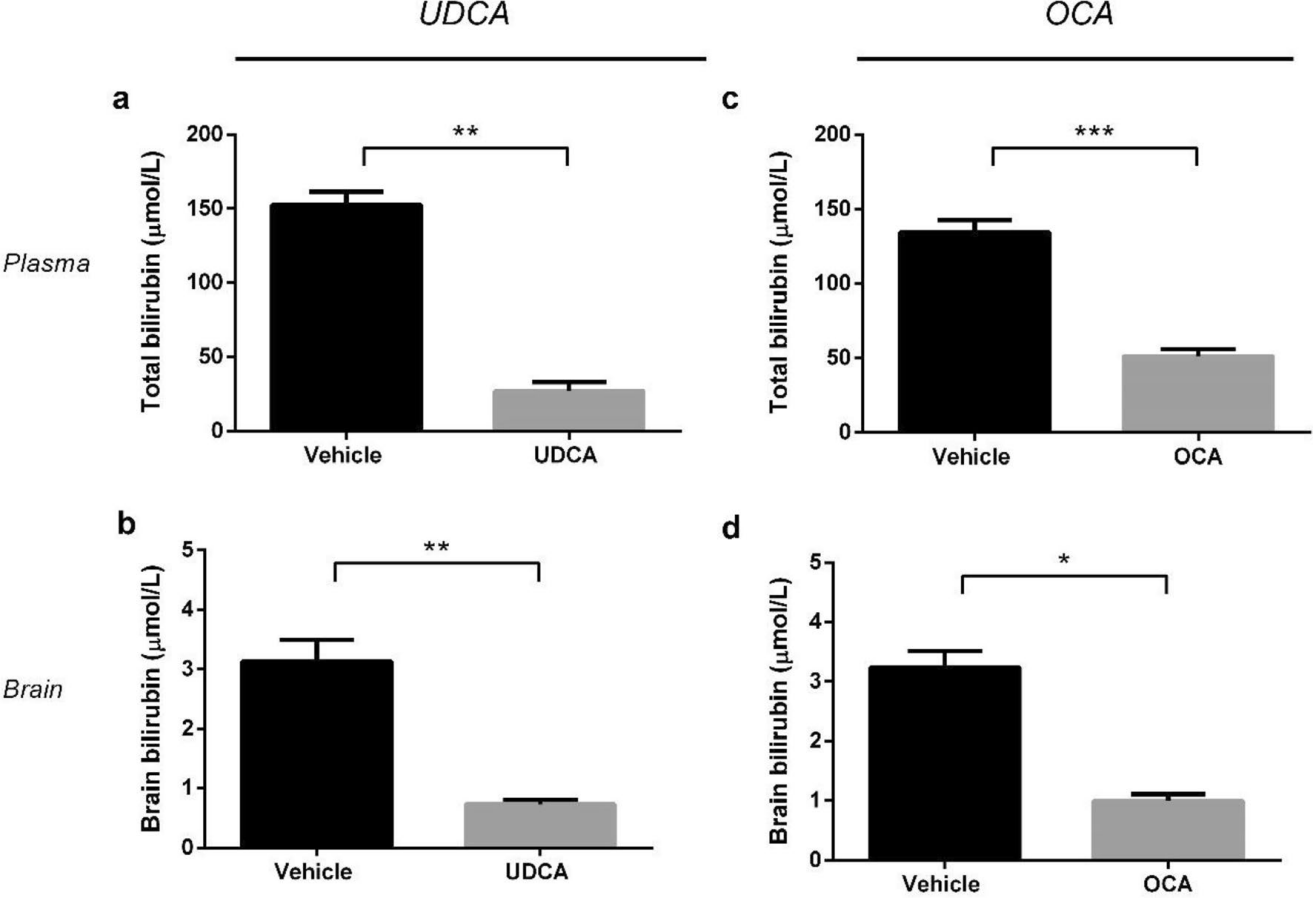
Effect of UDCA and OCA on plasma and brain bilirubin concentrations in neonatal hUGT1*1 mice. Levels of plasma and brain bilirubin in neonatal in hUGT1*1 mice after treatment with (**a**, **b**) UDCA (250 mg/kg/day, n = 6) or (**c**, **d**) OCA (50 mg/kg/day, n = 8). Concentrations of bilirubin in the brain tissue are expressed as µmol/L of tissue homogenate.

To determine the potential of anti-cholestatic drugs for the treatment of neonatal hyperbilirubinemia, hUGT1*1 pups were treated for five days (from P10-14) with UDCA (n = 6) or OCA (n = 8). UDCA (250 mg/kg/day) decreased total plasma bilirubin (TPB) by 82% (from 152 to 27 µmol/L, p < 0.01), whereas OCA (50 mg/kg/day) caused a 62% decrease (from 134 to 51 µmol/L, p < 0.001). Both UDCA and OCA also significantly decreased brain bilirubin concentrations by 77% (from 3.1 to 0.7 µmol/L, p < 0.01) and 69% (from 3.2 to 1.0 µmol/L, p < 0.03), respectively (Fig. 1), indicating a decreased neurotoxicity risk in these mice. The UDCA-induced TPB decrease was already apparent at 30 mg/kg/day (9%, p = 0.02) (n = 6), which is in the clinically used dose range of UDCA for preterm neonates. This decrease in TPB was also associated with decreased brain bilirubin content (18%), although this did not reach statistical significance (Fig. S1).

### Effect of UDCA and OCA on hUGT1A1 expression in liver and intestine

**Figure 2:**
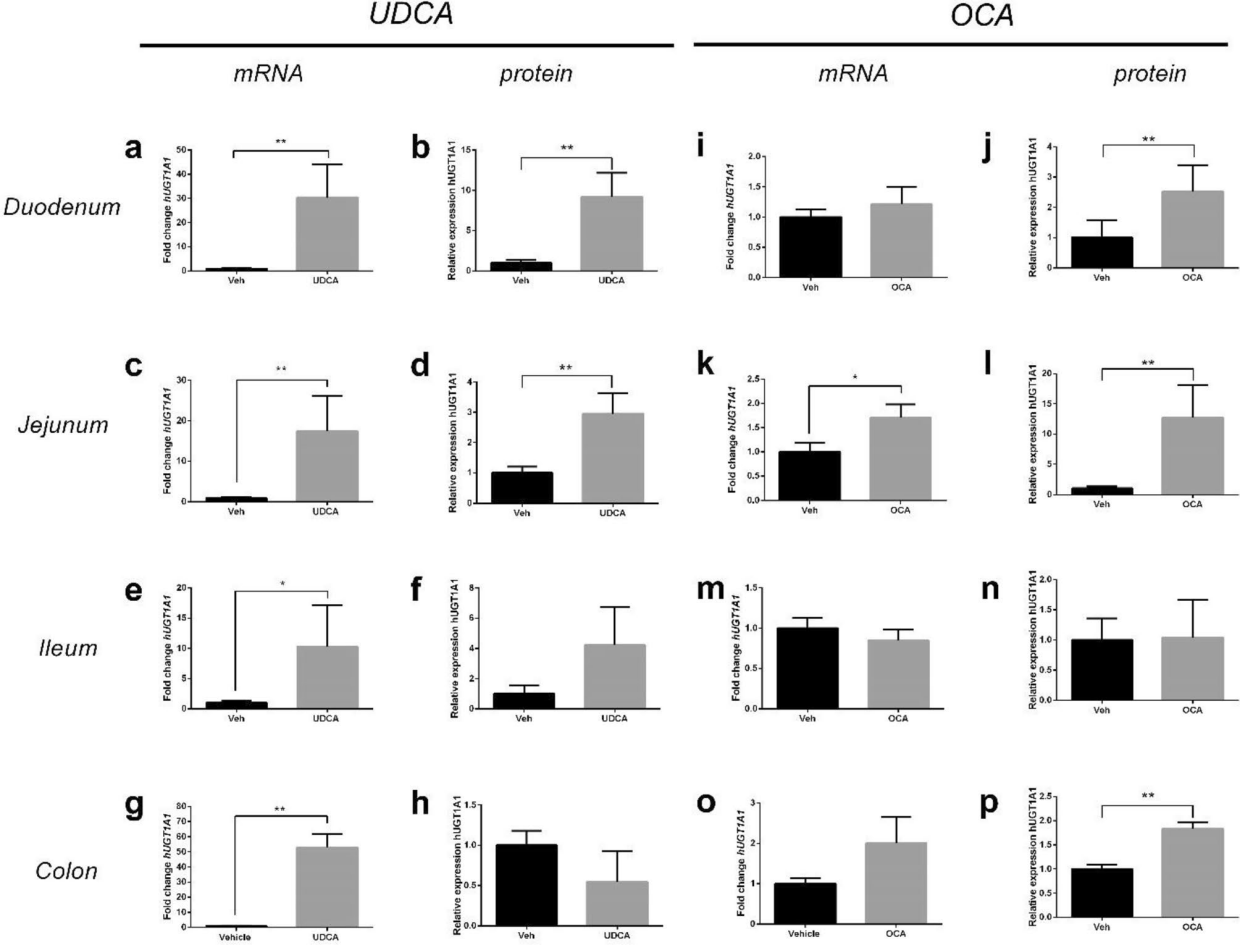
Effect of UDCA and OCA on intestinal hUGT1A1 mRNA and protein expression in neonatal hUGT1*1 mice. hUGT1A1 gene and protein expression after UDCA treatment in (**a**, **b**) duodenum, (**c**, **d**) jejunum, (**e**, **f**) ileum and (**g**, **h**) colon (n = 6). hUGT1A1 expression after OCA treatment in (**i**, **j**) duodenum, (**k**, **l**) jejunum, (**m**, **n**) ileum and (**o**, **p**) colon (n = 8).

To assess whether UDCA or OCA affected UGT1A1 expression in hUGT1*1 neonatal mice, we determined mRNA and protein levels in liver and intestine. Classically, the liver is regarded as the main organ responsible for bilirubin conjugation^24^. As expected, *UGT1A1* expression was not detectable in the liver of neonatal hUGT1*1 mice^17^, and was also not induced by UDCA or OCA (data not shown). In contrast to the liver, UDCA treatment strongly increased levels of *UGT1A1* mRNA in all intestinal sections (duodenum, jejunum, ileum and colon; Fig. 2a,c,e,g), whereas OCA treatment (Fig. 2i,k,m,o) only caused a 1.7-fold induction in jejunum (p < 0.05) (Fig. 2k). To assess whether the increased intestinal *UGT1A1* gene expression also resulted in increased protein expression, we performed Western blot analysis. In line with its mRNA levels, UGT1A1 protein levels were undetectable in liver whereas both UDCA (Figs. 2b,d,f,h; Fig. S2) and OCA (Figs. 2j,l,n,p; Fig. S2) treatment significantly increased hUGT1A1 protein levels in duodenum (both p < 0.01) and jejunum (both p < 0.01), but not in ileum. In colon, UGT1A1 protein expression was only induced after OCA treatment (p < 0.01) (Fig. 2p).

### FXR activation by UDCA and OCA

**Figure 3:**
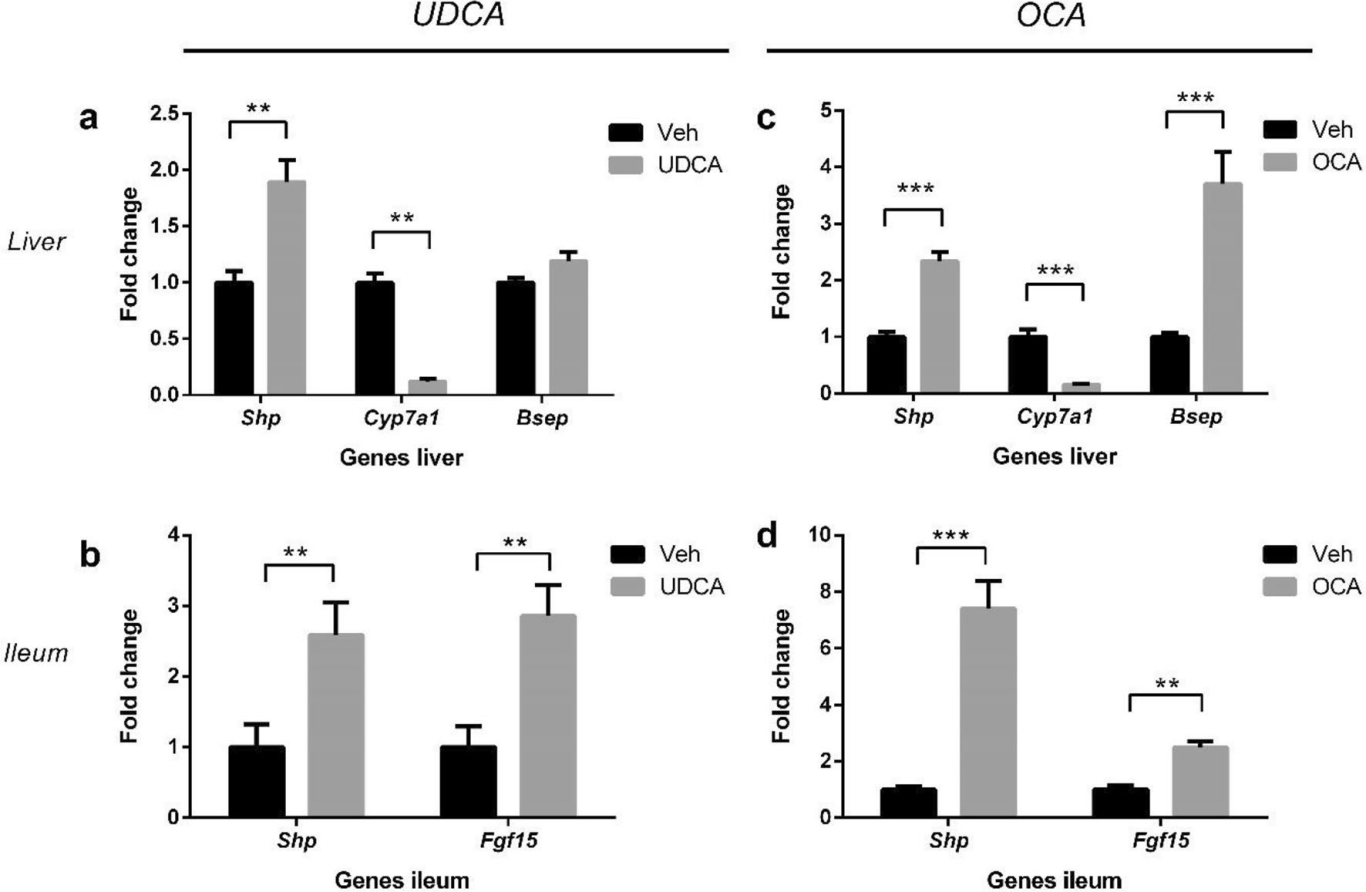
Effect of UDCA and OCA on expression of FXR-target genes in neonatal hUGT1*1 mice. mRNA levels of (**a**) hepatic and (**b**) ileal FXR target genes after vehicle or UDCA; (**c**) hepatic and (**d**) ileal FXR target genes after vehicle or OCA.

OCA is a selective FXR agonist, and therefore its actions can be potentially explained by activation of FXR. UDCA, on the other hand, is not an FXR agonist, but since it modulates BA homeostasis, it is possible that it can still indirectly affect FXR activity. To determine whether OCA and UDCA (directly or indirectly) caused FXR activation, we determined mRNA levels of the established FXR targets short heterodimer partner (*Shp, Nrob2*), cholesterol 7α-hydroxylase (*Cyp7a1*) and the bile salt exporting protein (*Bsep, Abcb11*) in liver and of *Shp* and fibroblast growth factor 15 (*Fgf15*) in ileum (Fig. 3). Both UDCA and OCA treatment resulted in hepatic and ileal FXR activation, although *Bsep* expression was not significantly affected by UDCA (Fig. 3).

**Figure 4:**
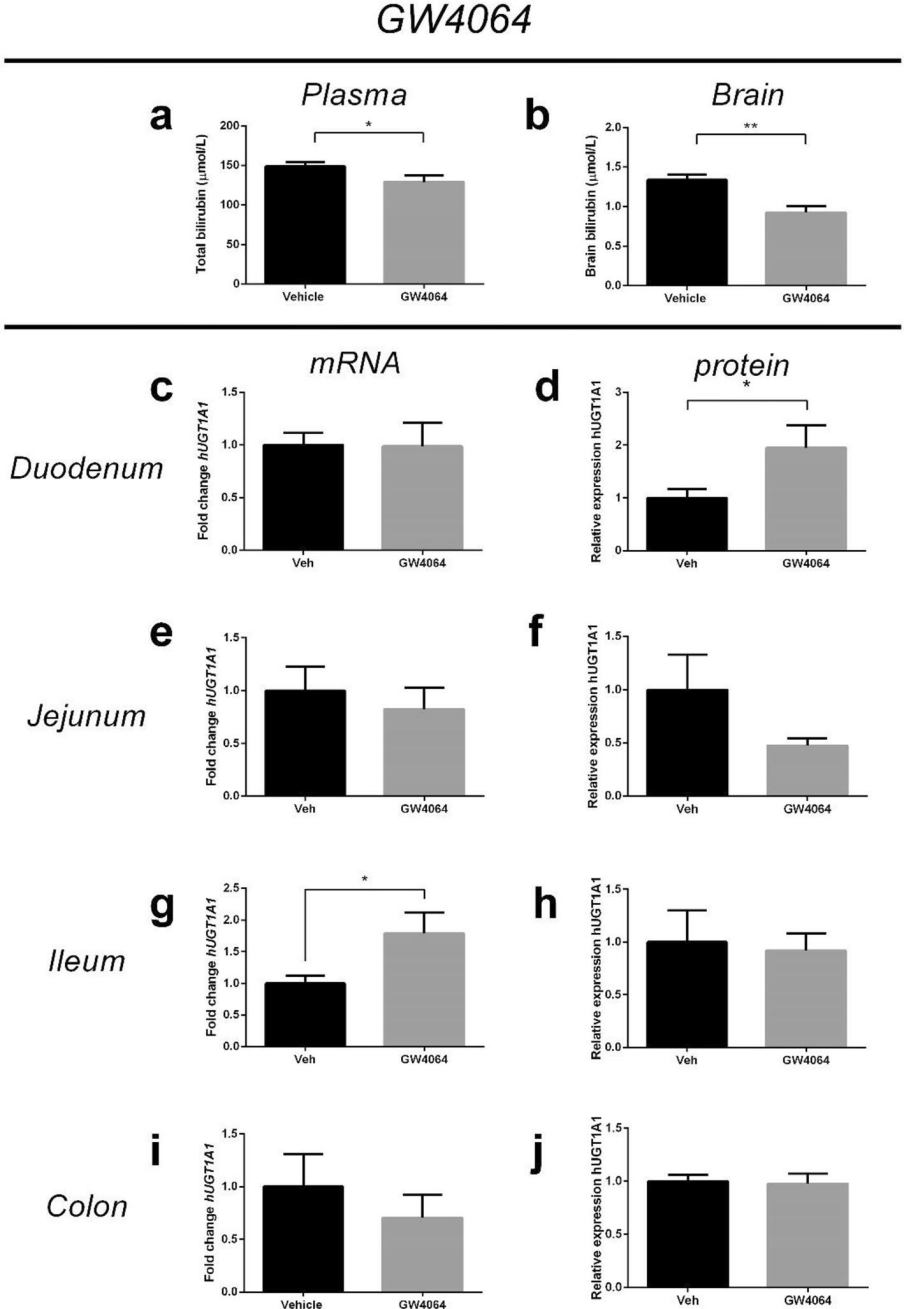
Effect of GW4064 on plasma and brain bilirubin and intestinal expression of hUGT1A1 in neonatal hUGT1*1 mice. Effect of GW4064 treatment (50 mg/kg/day) (n = 6) on (**a**) Total plasma bilirubin level; (**b**) Brain bilirubin levels; (**c**, **d**) mRNA and protein expression of hUGT1A1 in duodenum; and mRNA and protein expression of hUGT1A1 in (**e**, **f**) jejunum; (**g**, **h**) ileum and (**i**, **j**) colon.

Based on these findings, we hypothesized that the effects on UCB levels in plasma and brain could be mediated through FXR activation, either directly (OCA) or indirectly (UDCA). To further evaluate the potential involvement of FXR, we also tested the UCB lowering effects of the synthetic, non-BA, FXR-agonist GW4064, which is known to decrease BA production by suppression of CYP7A1. Treatment of hUGT1*1 pups with GW4064 (50 mg/kg/day) (n = 6) resulted in a similar gene expression pattern matching with FXR activation (Fig. S3). GW4064 also significantly reduced TPB by 13% (p < 0.05) and brain UCB by 31% (p < 0.01) (Fig. 4a,b), along with a twofold induction of UGT1A1 protein expression in duodenum (Fig. 4d). GW4064 also significantly increased *UGT1A1* mRNA expression in ileum, but this was not accompanied by increased protein expression (Fig. 4g,h). In jejunum and colon, GW4064 did not induce UGT1A1 (Fig. 4e,f,i,j). Collectively, these observations support a role for FXR in the UCB lowering effects of tested anti-cholestatic drugs.

### Contribution of UGT1A1 to the bilirubin-lowering effects of UDCA and OCA

**Figure 5:**
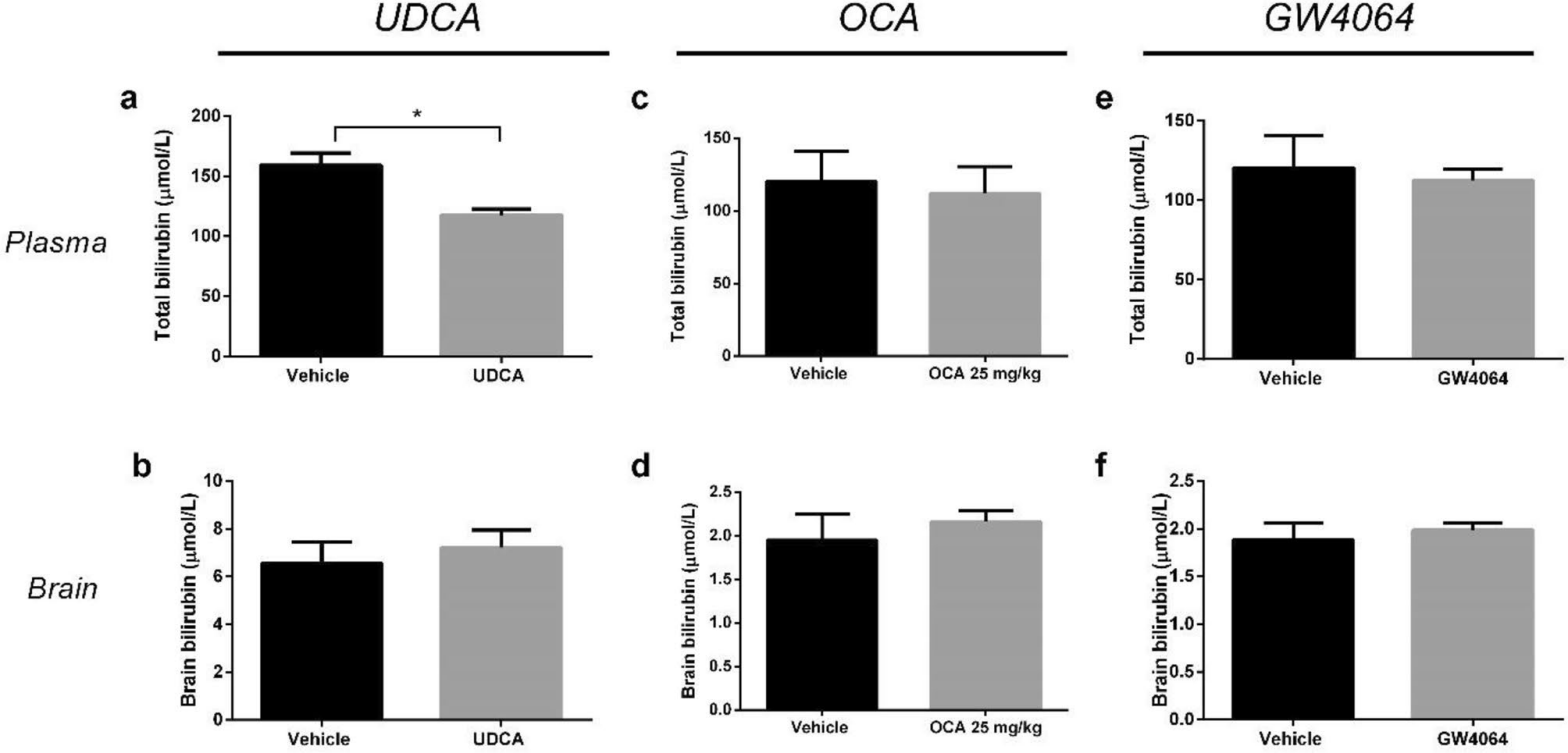
Effect of UDCA, OCA, and GW4064 on plasma and brain bilirubin in neonatal Gunn rats. Effect of (**a**, **b**) UDCA (250 mg/kg/day) (n = 6); (**c**, **d**) OCA (25 mg/kg/day) (n = 8), and (**e**, **f**) GW4064 (50 mg/kg/day) (n = 6) on total bilirubin levels in plasma and brain.

Although hUGT1*1 mice are dependent on hUGT1A1 expression for survival, this does not mean that the UDCA- and OCA-induced effects in these mice are necessarily or exclusively UGT1A1-mediated. To assess the role of UGT1A1, we therefore also tested both compounds in the *Ugt1a*-deficient Gunn rat model. In line with our previous observations in adult Gunn rats^13^, UDCA caused a significant decrease in plasma bilirubin levels of neonatal Gunn rats (26%, p < 0.05) whereas no effect was observed on brain bilirubin levels. In contrast, both OCA and GW4064 did not affect plasma or brain bilirubin levels in neonatal Gunn rats (Fig. 5). Unlike OCA and GW4064, UDCA treatment did not activate FXR in liver and intestine, indicating that the hypobilirubinemic effects of UDCA in Gunn rats were independent of FXR activation (Fig. S4).

## Discussion

In this study, we show that the therapeutic BAs UDCA and OCA effectively decrease plasma bilirubin in a humanized mouse model of neonatal hyperbilirubinemia. Both compounds also significantly reduced brain levels of bilirubin in these mice, highlighting their potential in the prevention of bilirubin-induced neurotoxicity. Mechanistically, we demonstrated that these effects are at least partially dependent on induction of intestinal UGT1A1 expression and on FXR activation.

Neonatal hyperbilirubinemia is caused by a limited capacity to metabolize bilirubin mainly due to delayed expression of UGT1A1. Since rodents express UGT1A1 at an earlier age than humans, they normally do not exhibit neonatal hyperbilirubinemia. For mice, it is known that a complete deficiency in *UGT1A1* expression is lethal within the first 4–7 postnatal days^17,20^, and that this condition can be rescued by bilirubin lowering treatments such as intraperitoneal albumin administration, *Ugt1a1* replacement gene therapy or by transgenic introduction of the human *UGT1A1* gene and its upstream regulatory region^20,25^. In hUGT1*1 mice, *UGT1A1* expression in neonates is almost exclusively intestinal, whereas in adults, it is expressed both in intestine and liver^17^. In human neonates, the intestinal *UGT1A1* expression and its potential contribution to neonatal bilirubin metabolism have never been studied, but there are several indications supporting the concept that intestinal bilirubin conjugation is also important in human physiology. Besides the liver, *UGT1A1* is also highly expressed in the small intestine. Already in the ’90 s, McDonnel et al*.* demonstrated *UGT1A1* expression and bilirubin conjugation activity in the human intestinal tract^26^. In adult human tissues, the intestinal conjugation of the UGT1A1 substrate estradiol even exceeds the hepatic conjugation capacity^27^. In premature human newborns, *UGT1A1* gene expression is repressed in the liver during the first postnatal days^18,19^. It is likely that intestinal *UGT1A1* expression is similar between *hUGT1A1* mice and human newborns with intestinal *UGT1A1* participating in the metabolism of serum bilirubin. However, this will be hard to establish both experimentally and ethically, in particular because healthy neonatal intestinal tissue is hardly available.

Both UDCA and OCA induced UGT1A1 mRNA and protein expression in duodenum and jejunum, which are also intestinal expression sites in human^26,28,29^. This induction could potentially be FXR-mediated, as we observed a significant bilirubin lowering effect by both OCA and GW4064, which are two structurally unrelated FXR ligands. Despite this, the bilirubin lowering effect by GW4064 and its induction of intestinal *Ugt1a1* were less prominent as compared to OCA. These differences are most likely explained by their different chemical structures and pharmacokinetic profiles. While OCA is a bile acid analogue which exhibits bile acid like pharmacokinetics (enterohepatic cycling), GW4064 is a non-steroidal (non-bile acid) synthetic FXR ligand with a relatively poor oral availability and which does not undergo enterohepatic cycling. These characteristics might result in a lower induction of intestinal *Ugt1a1* by GW4064.

Alternatively, the induction could be indirectly regulated, for example through activation of the pregnane-X-receptor (PXR, NR1I2), another member of the nuclear receptor superfamily. PXR activation results in a robust transcriptional induction of the *UGT1A1* gene while PXR itself can also be activated by BAs and transcriptionally regulated by FXR^30,31^. Besides regulation at the transcriptional level, it is also possible that hUGT1A1 induction is (partially) mediated at the posttranslational level. OCA, for example, did not increase mRNA levels of *hUGT1A1* in duodenum, but did induce the protein expression by 2.5-fold, which can potentially be explained by increased protein stability. Finally, FXR-independent mechanisms could play a role in the induction of hUGT1A1 by UDCA and OCA. To investigate this, however, it would require an *Fxr*-deficient humanized UGT1*1 mouse model, which is currently not available.

Our work also indicates that UDCA acts independently of *UGT1A1*, as UDCA treatment decreased TPB in *Ugt1a1*-deficient Gunn rats. This finding is in line with our previous studies in adult Gunn rats, in which UDCA decreased TPB by 21% after 7 days of treatment^13^. The TPB reduction in Gunn rat pups (-26%) is lower than the reduction observed in hUGT1*1 pups (-86%) and this can potentially be explained by a cumulative effect of UGT1A1-dependent and -independent UDCA effects in the latter.

There are several possible explanations for the hUGT1A1-independent effects. Upon treatment, UDCA becomes the major component of the BA pool, and thereby affects both the hepatic and intestinal BA composition^13^. Changes in intestinal BA composition could cause alterations in both hepatic and intestinal bilirubin metabolism. Hepatic conjugation is not possible in Gunn rats, and in previous work by our group, UDCA did not increase the biliary bilirubin concentration^13^. This makes hepatic interference of UDCA in bilirubin metabolism unlikely. However, in the same study, it was shown that UDCA caused a significant increase in fecal bilirubin, which indicates that UDCA either diminishes the intestinal bilirubin reabsorption or stimulates direct intestinal bilirubin excretion. Possibly, these mechanisms also play a role in the neonatal hUGT1*1 mice, but this could not be tested in the current study since collection of sufficient bile or feces was impossible at this young age.

The fact that OCA and GW4064 did not reduce TPB in Gunn rats, in contrast to hUGT1*1 mice, indicates that *hUGT1A1* expression is essential for the bilirubin-lowering effect. Another possible explanation for the absent effect of OCA could be the lower OCA dose used in rats (25/mg/kg/day) compared to mice (50 mg/kg/day). This approach was chosen because the high OCA dose was poorly tolerated in rats (data not shown). This lower dose could be responsible for the absent OCA effect, but not for the lack of effect by GW4064, which was administered at the same dose to Gunn rats and hUGT1*1 mice.

Although adult Gunn rats have been widely used to study neonatal hyperbilirubinemia, neonatal hUGT1*1 mice appear to be a more relevant model, since like human neonates, they have inducible hUGT1A1 expression that mediates the postnatal bilirubin course. Furthermore, it is a strictly neonatal model, which more closely mimics the human neonatal physiologic situation with a yet underdeveloped intestine, that is solely exposed to milk feeding. In adult humans and wild-type rodents, bilirubin levels barely depend on intestinal bilirubin metabolism and reabsorption, since in the presence of a fully-developed intestinal microbiome, bilirubin is effectively converted to urobilinogen and other derivates^32,33^. These metabolites are either not reabsorbed by the intestine, or efficiently excreted via the urine or bile after intestinal reabsorption^34^. However, in an inadequately colonized intestine, as typical for early neonatal period or after oral antibiotic treatment^32^, intestinal reabsorption profoundly contributes to the high TBP levels^35,36^. Therefore, it will be interesting to target intestinal bilirubin metabolism as a treatment strategy, especially in neonatal hyperbilirubinemia. UDCA and OCA alter the intestinal milieu and could thereby affect intestinal bilirubin handling, which could be especially beneficial during the neonatal period.

The *UGT1A1* gene in neonatal hUGT1*1 mice is transcriptionally regulated by nuclear receptors PXR, the constitutive-androstane receptor (CAR, NR1I3), and peroxisome proliferator-activated receptor α (PPAR α)^30,37,38^. Induction of *UGT1A1* was one of the underlying mechanisms of treatment with phenobarbital, a CAR agonist used as a standard treatment for neonatal hyperbilirubinemia before the introduction of phototherapy^39,40^ and used in Crigler-Najjar syndrome type 2^41^. *hUGT1* mice do not only contain the *UGT1A1* gene, but also the upstream regulatory region, including the distal regulatory PBREM that is activated by PXR and CAR^37^. This is important, since the *hUGT1A1* promoter regions are not conserved between species and transcriptional regulation of the gene differs between rodents and humans. By merely studying potential therapies in *Ugt1a1*-deficient models, such as Gunn rats, clinical effects could be missed, as we show for OCA, which did not decrease bilirubin in Gunn rats.

Bilirubin neurotoxicity is caused by brain bilirubin deposition (kernicterus) and therefore, strongly depends on the maturity and permeability of the blood–brain barrier^36^. In homozygous and heterozygous Gunn rats, we observed that brain bilirubin levels are significantly higher in neonates as compared to (young) adults and that the ratio between brain and plasma bilirubin is higher in neonates than in adults (data not shown). This phenomenon has also been described in piglets, in which the ratio between brain bilirubin and free bilirubin in plasma decreases fivefold between P2 and P14^42^. Therefore, we used neonatal animals to study the effects of UDCA and OCA on brain bilirubin deposition and the consequent neurotoxicity risk.

Interestingly, we show that UDCA only decreased plasma, but not brain bilirubin levels in neonatal Gunn rats, whereas it decreased both plasma and brain bilirubin levels in neonatal hUGT1*1 mice. This phenomenon could have several explanations. Firstly, a more profound decrease in plasma bilirubin levels might be needed to induce a decrease in brain bilirubin in the Gunn rat. However, in the hUGT1*1 mice, the relative decrease in brain bilirubin seemed to follow closely the corresponding decrease in plasma. This possibly results from the fact that *UGT1A1* is expressed in brain of hUGT1*1 mice. Although bilirubin conjugation in the brain has never been demonstrated, UGT1A1 was previously shown to mediate estradiol conjugation in the brain^43^. Therefore, brain UGT1A1 could potentially contribute to local bilirubin biotransformation and export. Secondly, bilirubin is exported from the brain by species-specific transporters such as Mdr1a P-glycoprotein (Abcb1a), which are differentially regulated between mice and rats^44^. Potentially, UDCA may upregulate a mouse transport protein that is not present or not regulated similarly in Gunn rats.

In conclusion, we show that both UDCA and OCA strongly decrease bilirubin in plasma and brain of neonatal hUGT1*1 mice. Mechanistically, our work indicates that intestinal, rather than hepatic hUGT1A1, plays an important role in these effects. Furthermore, it suggests that OCA acts via hUGT1A1-dependent mechanisms, whereas UDCA also has partial hUGT1A1-independent effects. Both UDCA and OCA are FDA-approved drugs, and UDCA is even already approved for neonatal use, making them readily available for clinical studies. Both compounds could potentially offer a prevention strategy for neonatal hyperbilirubinemia.

## Methods

### Animals

The generation of humanized UGT1*1 mice (> 99% C57BL/6J background) has been described previously^17^. Before and during the experiments, all pups were housed with their mother with ad libitum access to breast milk. Dams had ad libitum access to water and food (Picolab Rodent Diet 20, 5053 breeding diet, (Labdiet, St. Louis, MO). The litters were kept in an environmentally controlled facility with a 12 h day/night cycle. Gunn rats (originally obtained from Rat Resource Center (Columbia, MO) were bred at the University Medical Center Groningen (UMCG) animal facility. Dams were fed ad libitum with RM03 breeding chow (Special Diet Services, Essex, UK).

### Animal procedures

Pups (both male and female) within every litter were randomized to receive vehicle (ORA-plus oral suspension solution, Allegan, MI) or vehicle with either UDCA (Sigma-Aldrich, St. Louis, MO), OCA (MedChemExpress, South Brunswick, NJ), or GW4064 (Cayman Chemicals, Ann Arbor, MI). UDCA was given at 250 mg/kg/day once daily via oral gavage. Both OCA and GW4064 were given at a dose of 50 mg/kg/day, divided over 2 doses daily. Also, hUGT1*1 mice were treated with the clinically recommended UDCA dose for neonates (30 mg/kg/day in 2 doses per day). All mice were treated daily from P10-14. Mice were sacrificed within 6 h after the last dose on P14 by decapitation under isoflurane anesthesia. Blood was collected in EDTA-coated capillaries from the trunk and stored on ice in the dark upon centrifugation and plasma was stored at -80° C under argon until analysis. Liver and spleen were snap-frozen upon harvesting and subsequently pulverized. The intestine was flushed with ice-cold PBS before snap freezing. Brains were snap frozen and stored in amber-colored tubes at – 80 °C. Since OCA is associated with hepatotoxicity, we tested its effect on plasma activities of ALT and AST. OCA treatment induced moderately increased activities of plasma ALT but had no significant effect on plasma AST, suggesting that hepatotoxicity under these conditions was limited (Fig. S5).

Gunn rat pups were treated with UDCA (250 mg/kg/day) divided over 2 daily doses from P7-14 or with OCA (25 mg/kg/day) or GW4064 (50 mg/kg/day) from P10-14 in one daily dose. Gunn rat pups were terminated on P14 within 6 h after the last dose by cardiac puncture under isoflurane anesthesia and tissues collected and stored as described. All experiments with hUGT1*1 mice were performed at UCSD and approved by the local Animal Ethics Committee of University of California San Diego. All experiments with Gunn rats were performed at the UMCG with the approval of the local Ethics Committee for Animal Experiments of the University of Groningen. All experiments were performed in accordance with relevant guidelines and regulations (including laboratory and biosafety regulations).

### Bilirubin analysis

TPB analysis in hUGT1*1 mice was performed using a UNISTAT Bilirubinometer (Reichert Technologies, Depew, NY). TPB analysis in Gunn rats was performed using the Bilirubin Total Gen 3 kit (Roche Diagnostics, Rotkreuz, Switzerland) on a Roche/Hitachi Cobas 501 Analyzer (Hitachi, Tokyo, Japan). Analysis of bilirubin in the brain tissue was performed as described previously^45^. Briefly, approximately 100 mg of homogenized brain was mixed with 50 μL of 5 μM internal standard mesobilirubin (Frontier Scientific, Logan, UT) and homogenized with glass rod. Bilirubin from this mixture was extracted into methanol/chloroform/hexane (10/5/1, v/v/v). Afterwards, the resulting phase was mixed with n-hexane and carbonate buffer (pH 10), vortex-mixed and centrifuged. Fifty μL of the resulting polar droplet was loaded onto reverse C-8 column (Luna 3 µm, 150 × 4.6 mm, Phenomenex, Torrance, CA) and the amount of bilirubin was determined on HPLC (Agilent 1200 DAD, Agilent, Santa Clara, CA). Concentrations of bilirubin in the brain tissue were expressed in µmol/L of tissue homogenate.

### Gene expression analysis

Total RNA was isolated from the liver, small intestine (duodenum, jejunum, ileum) and colon of mice using Trizol (Life Technologies, USA) and reverse transcribed into cDNA using iSCRIPT (Bio-Rad). For quantitative PCR (qPCR), cDNA was amplified using Hi-ROX SensiMix™ SYBR green (Bioline, London, UK) or Taqman fast mix (Applied Biosystems, Foster City, CA) using the Quant Studio Real-Time qPCR (Applied Biosystems) with gene-specific primers (Table S1). Each sample was quantified using a standard curve of the pooled samples and normalized for cyclophilin.

### Protein analysis

For immunoblot analysis, tissue lysates were obtained using RIPA lysis buffer (Thermo-Fischer Scientifc, Waltham, MA). After determination of protein concentration, 30 μg protein was loaded on a pre-cast NuPAGE BisTris gel (Thermofisher, Waltham, MA). The resolved protein was transferred onto a 45 µm PVDF membrane (Merck Millipore, Burlington, MA). After blocking, the membrane was incubation with hUGT1A1 primary antibody (ab170858, Abcam, Cambridge, UK) and GAPDH (sc-32233, Santa Cruz Biotechnology, Dallas, TX) and secondary antibody (anti-mouse IgG HRP-linked 7076S and anti-rabbit IgG HRP-linked 7074, Cell Signaling, Danvers, MA) detected by the Clarity Western ECL Substrate (Biorad, Hercules, CA) and visualized using the Bio-Rad ChemiDoc imaging system.

### Statistical analysis

GraphPad Prism 5.00 software package (GraphPad Software, San Diego, CA) was used to perform statistical analysis. Since the that the data was not normally distributed, significance was determined using nonparametric Mann Whitney U-test. All values are given as means ± standard error unless stated otherwise. Significance is indicated as *P < 0.05, **P < 0.01, ***P < 0.001.

## Supplementary Information

Supplementary Information.

## Data Availability

The data generated in the current study are available from the corresponding author upon request.
