# Supplementary material for: Potential of therapeutic bile acids in the treatment of neonatal Hyperbilirubinemia
Source: Sci Rep. 2021 May 27;11:11107. doi: 10.1038/s41598-021-90687-5 (PMC8160219; doi:10.1038/s41598-021-90687-5)
Supplement: Supplementary file 1 — Supplementary Information. [file 41598_2021_90687_MOESM1_ESM.docx]

**POTENTIAL OF THERAPEUTIC BILE ACIDS IN THE TREATMENT OF NEONATAL HYPERBILIRUBINEMIA**

Lori W.E. van der Schoor^1,2^, Henkjan J. Verkade^1,2^, Anna Bertolini^1^, Sanne de Wit^1^, Elvira Mennillo^3,^ , Eva Rettenmeier^3^, André A. Weber^3^, Rick Havinga^1^,

Petra Valášková^4^, Jana Jašprová^4^, Dicky Struik^1^, Vincent W. Bloks^1^, Shujuan Chen^3^, Andrea B. Schreuder^1,2^, Libor Vítek^4^, Robert H. Tukey^3#^ and Johan. W. Jonker^1#^

**Supplemental material**

**Table S1: qPCR primer sequences**

| **Gene** | **Forward primer 5’-3’** | **Reverse primer 5’-3’** |
| --- | --- | --- |
| **Mouse** | | |
| *Cyclophilin (Sybr)* | ATG GTC AAC CCC ACC GTG T | TTT CTG CTG TCT TTG GAA CTT TGT C |
| *Cyclophilin (Taqman)* | CAG ATC GAG GGA TCG ATT CAG | TCA CCA CTT GAC ACC CTC ATT C |
| hUGT1A1 | AAC AAG GAG CTC ATG GCC TCC | GTT CGC AAG ATT CGA TGG TCG |
| *Fxr (Nr1h4)* | CGC TGA GAT GCT GAT GTC TTG | CCA TCA CTG CAC ATC CCA GAT |
| *Shp(Nr0b2)* | AAG GGC ACG ATC CTC TTC AA | CTG TTG CAG GTG TGC GAT GT |
| *Cyp7a1* | CAG GGA GAT GCT CTG TGT TCA | AGG CAT ACA TCC CTT CCG TGA |
| *Bsep(Abcb11)* | CTG CCA AGG ATG CTA ATG CA | CGA TGG CTA CCC TTT GCT TCT |
| *Fgf15* | GCC ATC AAG GAC GTC AGC A | CTT CCT CCG AGT AGC GAA TCA G |
| **Rat** | | |
| Cyclophilin (Taqman) | CAG ATC GAG GGA TCG ATT CAG | TCA CCA CTT GAC ACC CTC ATT C |
| hUGT1A1 | n.a. | n.a. |
| *Fxr(Nr1h4)* | CGC TGA GAT GCT GAT GTC TTG | CCT TCA CTG CAC ATC CCA GAT |
| *Shp(Nr0b2)* | ACC TGC AAC AGG AGG CTC ACT | TGG AAG CCA TGA GGA GGA TTC |
| *Cyp7a1* | CAG GGA GAT GCT CTG TGT TCA | AGG CAT ACA TCC CTT CCG TGA |
| *Bsep(Abcb11)* | TTCTTGCAAATTCCGCTGCC | AGACCACCCTGAAAACGTGG |
| *Fgf15* | GCC ATC AAG GAC GTC AGC A | CTT CCT CCG AGT AGC GAA TCA G |
| *ApoC3* | CCA AGA CGG TCC AGG ATG C | ACT TGC TCC AGT AGC CTT TCA GG |

***
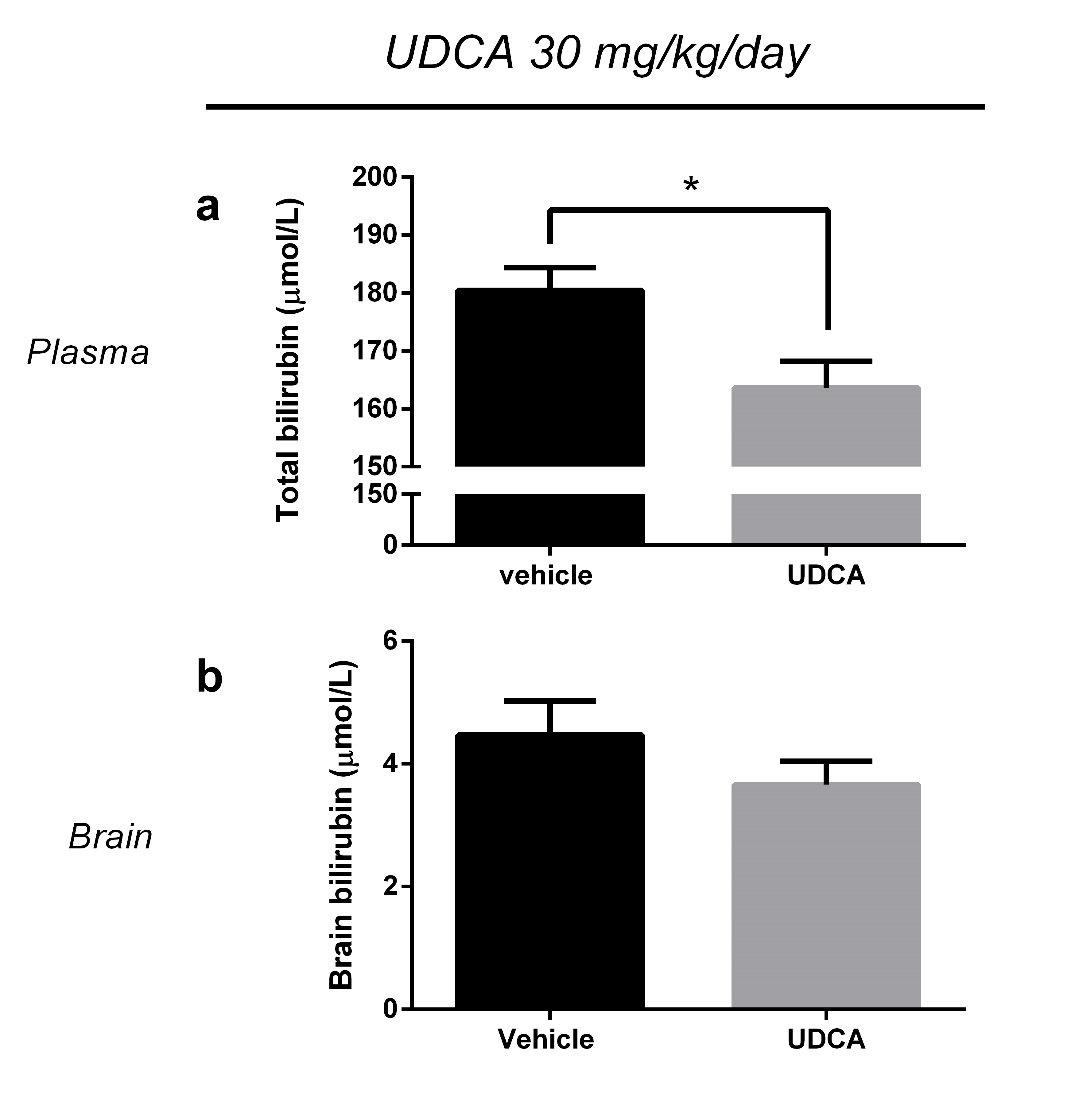
***

***Figure S1: Effect of a clinically relevant dose of UDCA on plasma and brain bilirubin. a)*** *Total plasma bilirubin and brain bilirubin* ***b)*** *after treatment with vehicle or UDCA (30 mg/kg/day). Concentrations of bilirubin in the brain tissue were expressed in μmol/L of tissue homogenate.*

*
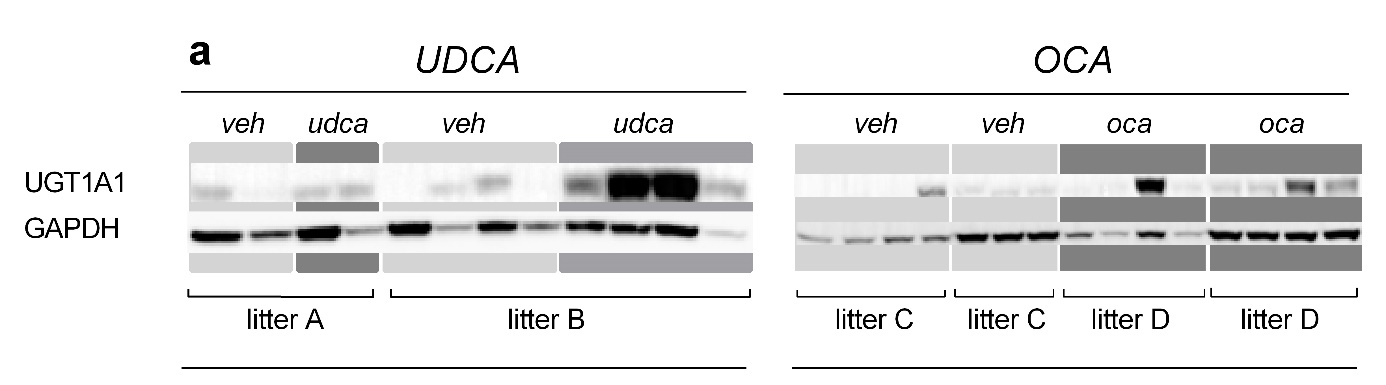
*

**b**


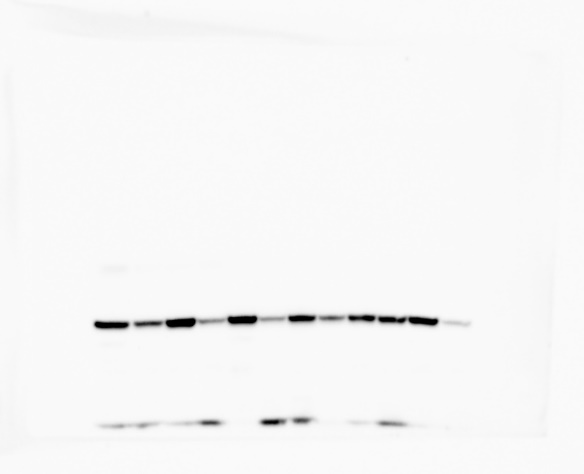

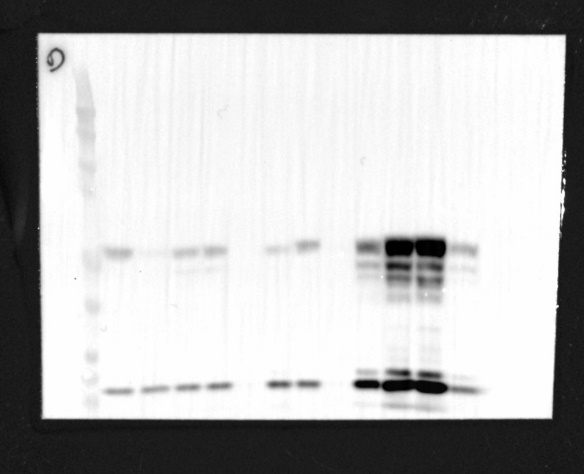


**c *UDCA duodenum hUGT1A1***

**d *UDCA duodenum GAPDH***


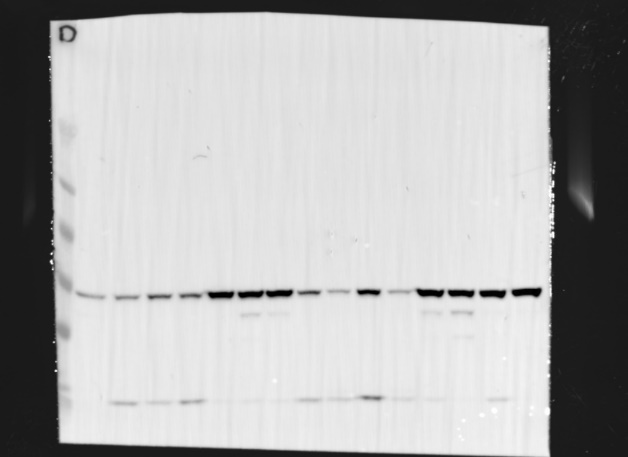

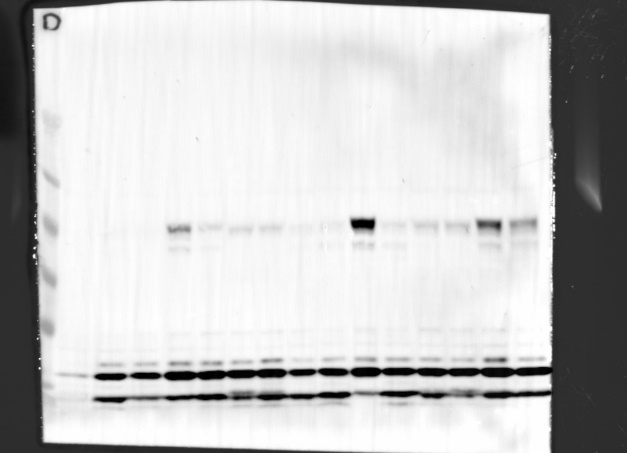


**f  *OCA duodenum GAPDH***

**e  *OCA duodenum hUGT1A1***

***Figure S2: Western blot analysis of hUGT1A1 and GAPDH in duodenum****. Western blots of hUGT1A1 and GAPDH in neonatal hUGT1*1 mice after* ***a)*** *treatment with vehicle or UDCA and* ***b)*** *treatment with vehicle or OCA. Raw western blots of* ***c)*** *hUGT1A1 and* ***d)*** *GAPDH in duodenum after UDCA treatment and* ***e)*** *hUGT1A1 and* ***f)*** *GAPDH in duodenum after OCA treatment.*

***
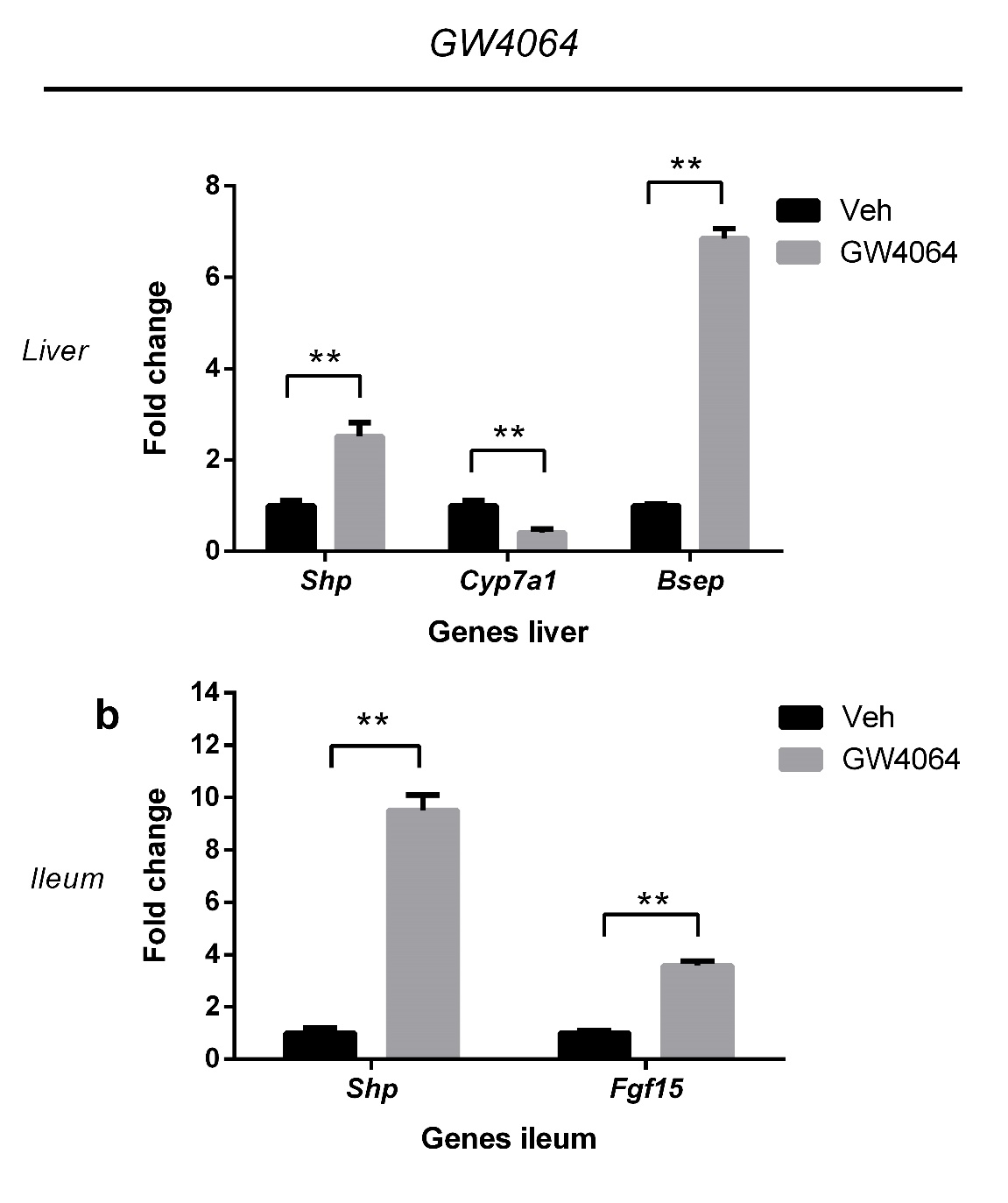
***

***Figure S3. mRNA expression of FXR target genes after GW4064 treatment in neonatal hUGT1*1 mice.*** *Gene expression of* ***a)*** *hepatic and* ***b)*** *ileal FXR target genes after treatment with vehicle or GW4064 in hUGT1*1 mice.*

**
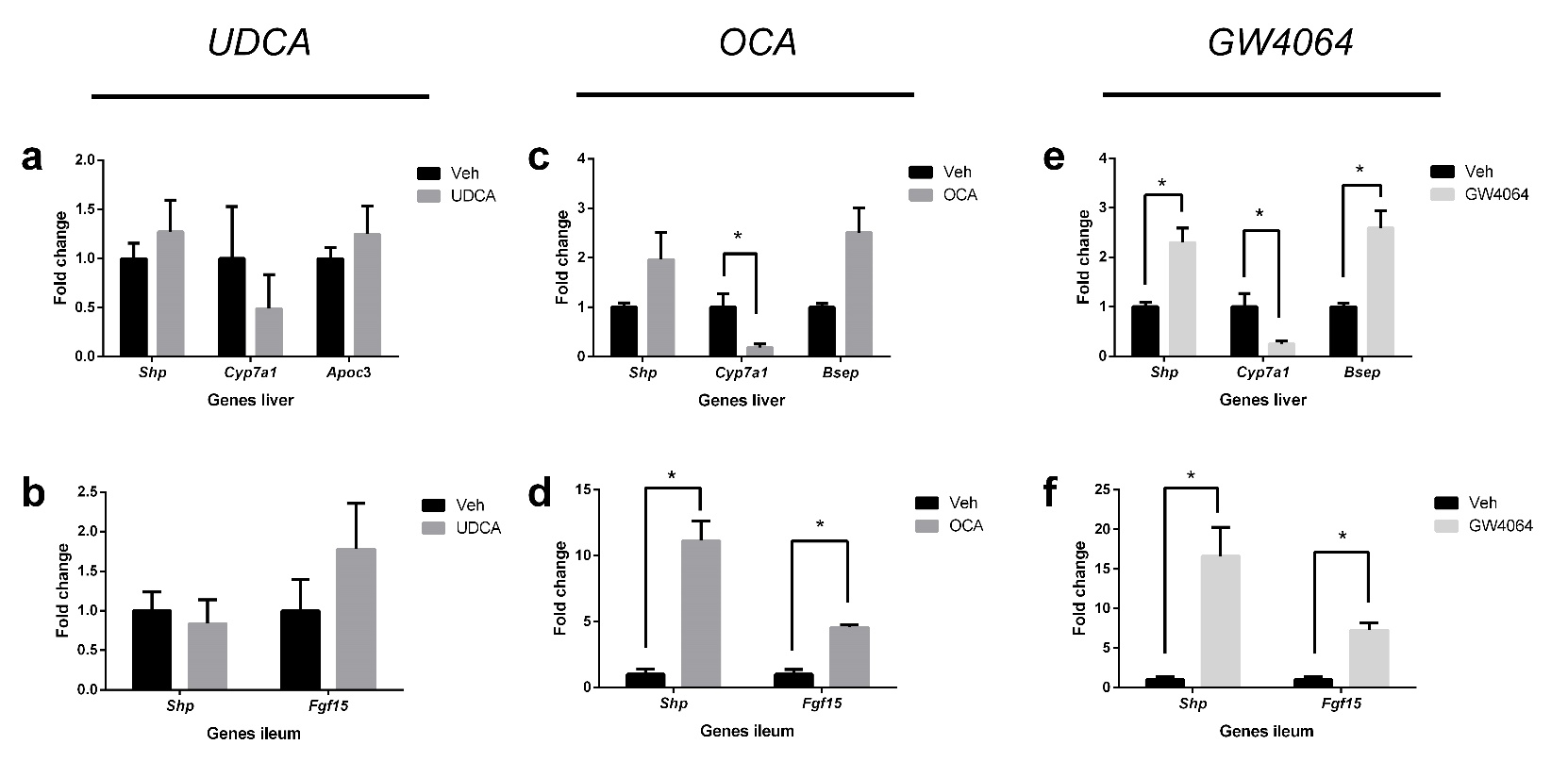
*Figure S4: mRNA expression FXR target genes in neonatal Gunn rats after UDCA, OCA or GW4064.*** *mRNA levels of* ***a)*** *hepatic and* ***b)*** *ileal FXR target genes after vehicle or UDCA in Gunn rats (n=5).* *mRNA levels of* ***c)*** *hepatic and* ***d)*** *ileal FXR target genes after vehicle or OCA (25 mg/kg/day) (n=5). mRNA levels of* ***e)*** *hepatic and* ***f)*** *ileal FXR target genes after vehicle or GW4064 (50 mg/kg/day) (n=4).*

***
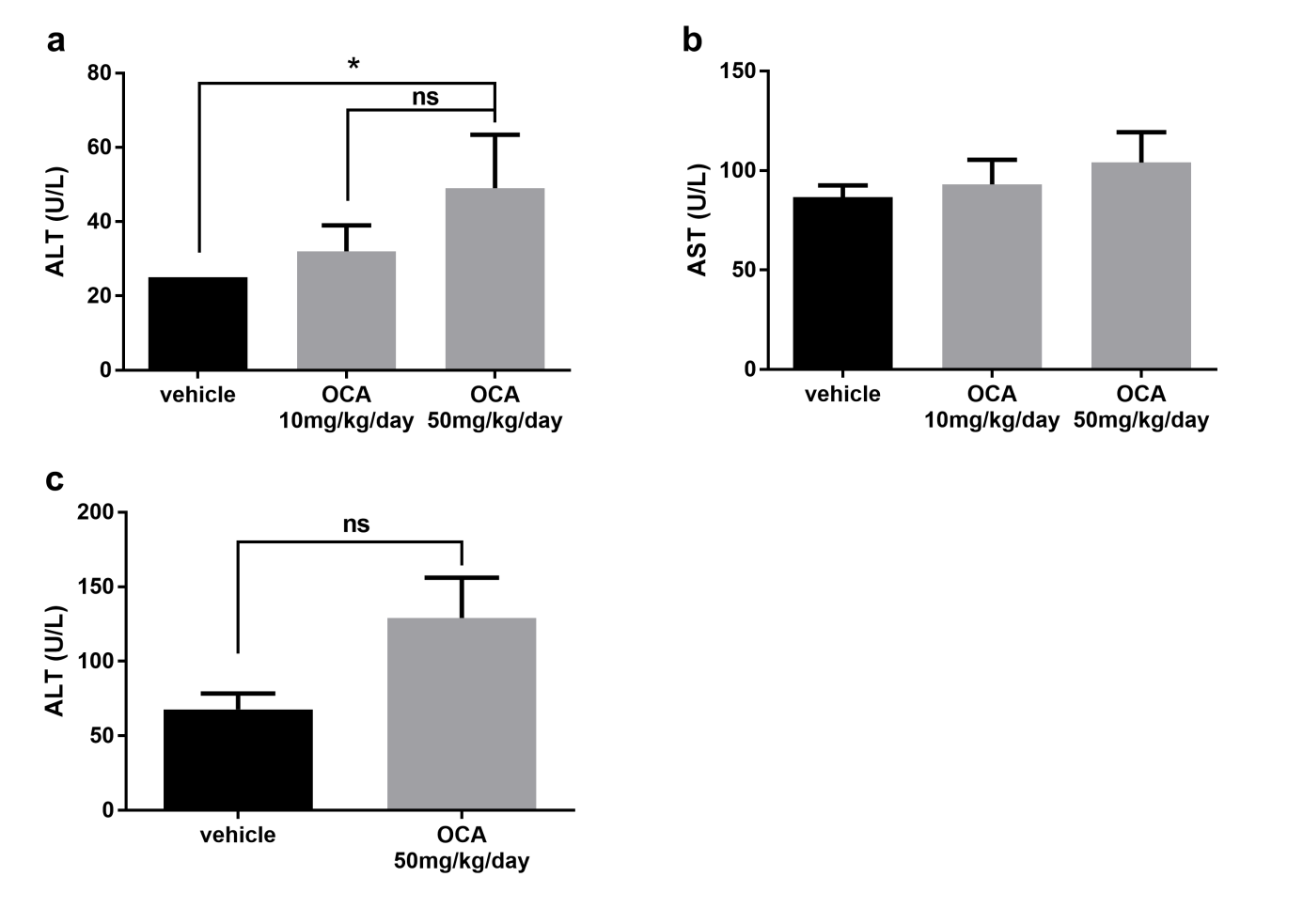
***

***Figure S5: Effect of OCA treatment on plasma activities of ALT and AST in neonatal mice.*** *Effect of OCA (10 and 50 mg/kg/day) treatment on plasma activities of* ***a)*** *ALT and* ***b)*** *AST in wild type neonatal mice (n = 5); Effect of OCA (50 mg/kg/day) treatment on plasma activities of ALT in neonatal hUGT1*1 mice (n = 3).*
